# Beyond Beneficial Margins: Four Mechanisms Linking Border Vegetation to Pest Dynamics

**DOI:** 10.3390/biology15090697

**Published:** 2026-04-29

**Authors:** Jorge F. M. Cardoso, Fabiane M. Mundim

**Affiliations:** 1 Department of Biology, Utah State University, Logan, UT 84322, USA; jorge.cardoso@usu.edu; 2 Ecology Center, Utah State University, Logan, UT 84322, USA

**Keywords:** weed, field margins, reservoir habitats, multitrophic interactions, insect pests, soil-associated arthropods

## Abstract

Vegetated field borders are widely promoted as tools to enhance biodiversity and strengthen biological control in agroecosystems. However, their role in pest dynamics remains conceptually fragmented and empirically inconsistent. Here, we develop a unified framework explaining how crop border vegetation influences pest populations through four interlinked ecological mechanisms. First, borders act as host reservoirs and selective filters, providing alternative hosts and overwintering habitat that enhance pest persistence across crop cycles. Second, borders modify pest colonization dynamics by shaping movement, aggregation, and host-location behavior at crop edges. Third, borders restructure multitrophic networks, simultaneously supporting natural enemies, alternative prey, vectors, and pathogens, generating nonlinear effects on pest suppression. Fourth, repeated disturbance and management function as selective filters, determining which plant functional groups dominate borders and, consequently, which pest and natural enemy communities are maintained. To ground this framework, we conduct a structured synthesis of published empirical and conceptual studies on crop-border vegetation, including weed and arthropod surveys, and classify them according to the proposed mechanisms. Our synthesis reveals a strong emphasis on multitrophic effects, whereas colonization processes and disturbance filtering are comparatively underexplored. Across mechanisms, plant identity and dominance structure consistently emerge as stronger predictors of pest outcomes than species richness alone. We argue that borders are not inherently beneficial or harmful but function as selectively structured ecological interfaces shaped by management history and species composition. By integrating temporal persistence, spatial behavior, network interactions, and anthropogenic filtering, our framework provides a predictive basis for IPM-oriented design of field borders, enabling management strategies that reduce pest carryover, disrupt colonization pathways, and enhance biological control while maintaining ecosystem services. This article is part of the theme issue “*The Biology, Ecology, and Management of Plant Pests*”.

## Simple Summary

Field borders, the strips of vegetation along the edges of crop fields, are often promoted as beneficial for supporting pollinators and natural enemies of pests. However, their effects on pest populations are not always consistent and can sometimes even increase pest problems. In this study, we developed a framework to explain how border vegetation influences pest populations through four main processes: by helping pests survive between growing seasons, influencing how they move into crops, changing interactions with predators and diseases, and being shaped by farm management practices such as mowing or herbicide use. We used existing research and a testing field survey of border vegetation and associated pests to evaluate these processes. We show that field borders are not just random collections of plants. Instead, a few dominant plant species often have the strongest influence on whether borders increase or reduce pest presence. This means that plant composition matters more than simply how many plant species are present. We conclude that managing field borders based on plant identity and composition can improve pest control while still supporting other ecological benefits. This work provides a practical approach for designing field borders that support more sustainable agriculture.

## 1. Introduction

**Figure 1:**
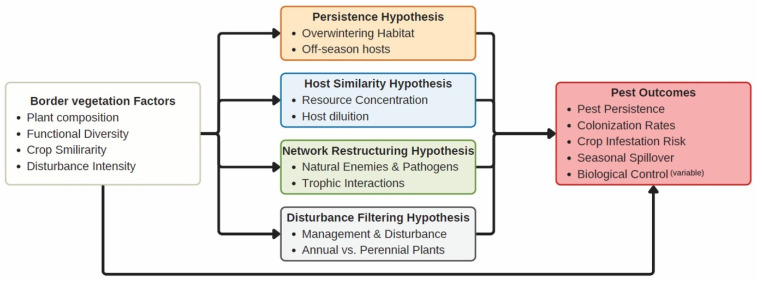
Conceptual schematic of four hypotheses explaining how field border vegetation influences pest dynamics in agroecosystems. Border vegetation characteristics (composition, diversity, crop similarity, and disturbance) influence pest populations through four interacting mechanisms: persistence via seasonal host continuity, host similarity effects on resource concentration or dilution, restructuring of multitrophic interaction networks, and disturbance-driven environmental filtering. These mechanisms jointly regulate pest persistence, colonization, and spillover into crops.

Weeds are typically defined as plants that interfere with human goals, particularly agricultural production, where they compete with crops for light, nutrients, water, and space, reducing yields and increasing management costs. However, their ecological roles extend beyond direct competitive interactions and include important contributions to agroecosystem functioning. Many weed species serve as alternative hosts for insect herbivores, reservoirs for plant pathogens, and modulators of soil microbial communities, thereby contributing to agroecosystem networks that shape plant health and pest dynamics. While weeds within fields are intensively managed [1,2,3], vegetated field borders are often dominated by unmanaged or semi-managed weed communities. These border habitats are increasingly valued for supporting ecosystem services such as pollination and biological control [2,4,5], but their composition and ecological similarity to crops also position them as potential nodes in pest and pathogen dynamics across agricultural landscapes (Figure 1).

Weeds occupy an ecological paradox in agroecosystems. Although they are primary targets of suppression within crop fields, they also contribute substantially to biodiversity in non-crop habitats such as field margins and borders [4,6,7,8]. These border environments typically experience lower disturbance intensity and greater light availability than crop interiors, conditions that favor the establishment of diverse plant assemblages [4]. Ecological theory predicts that species coexistence in such environments is promoted by spatial and temporal variation in resource availability, which allows species with differing trait syndromes to partition limiting resources [9,10,11,12,13]. Coexistence theory further indicates that stabilizing niche differences (e.g., resource partitioning, temporal storage effects) and fitness differences jointly determine local diversity [14,15,16,17]. In border habitats, variability in light, soil nutrients, disturbance intensity, and microclimate creates fine-scale environmental heterogeneity that promotes the persistence of species with divergent functional strategies, including shallow- versus deep-rooted species, early- versus late-flowering taxa, and annual ruderal versus perennial stress-tolerant plants [9,18,19,20]. Such heterogeneity increases the likelihood that border plant assemblages include a broad range of structural forms and phenologies, thereby expanding the trophic and microhabitat resources available to pollinators, herbivores, natural enemies, and decomposers [21,22,23,24,25]. As a result, border habitats often support heterogeneous plant communities that provide food and habitat for organisms associated with ecosystem services, a biodiversity-supporting role that has led to the widespread promotion of vegetated borders as tools for ecological intensification (Figure 1).

However, this ecosystem-service framing obscures an important demographic and spatial ecological process: in highly managed agroecosystems, recolonization from field borders and ruderal habitats increasingly shapes weed community composition within fields [7,26,27]. From a spatial population perspective, borders function as demographic source habitats within a metapopulation network of weed species [26,28]. Repeated disturbance, tillage, and herbicide filtering inside fields elevate local extinction rates for many species, particularly those with slower life histories or limited resistance traits ([28,29]; Figure 1). Field margins and ruderal habitats can offset these losses by supplying propagules via seed rain, vegetative spread, and dispersal along machinery tracks or water flow, effectively rescuing local populations from extinction [30,31]. Although persistent seedbanks buffer some management effects, the balance between local extinction and recolonization increasingly determines in-field diversity. Consequently, weed communities within crops may reflect landscape-level dispersal dynamics and source-sink processes as much as in-field competition [28,32].

**Figure 2:**
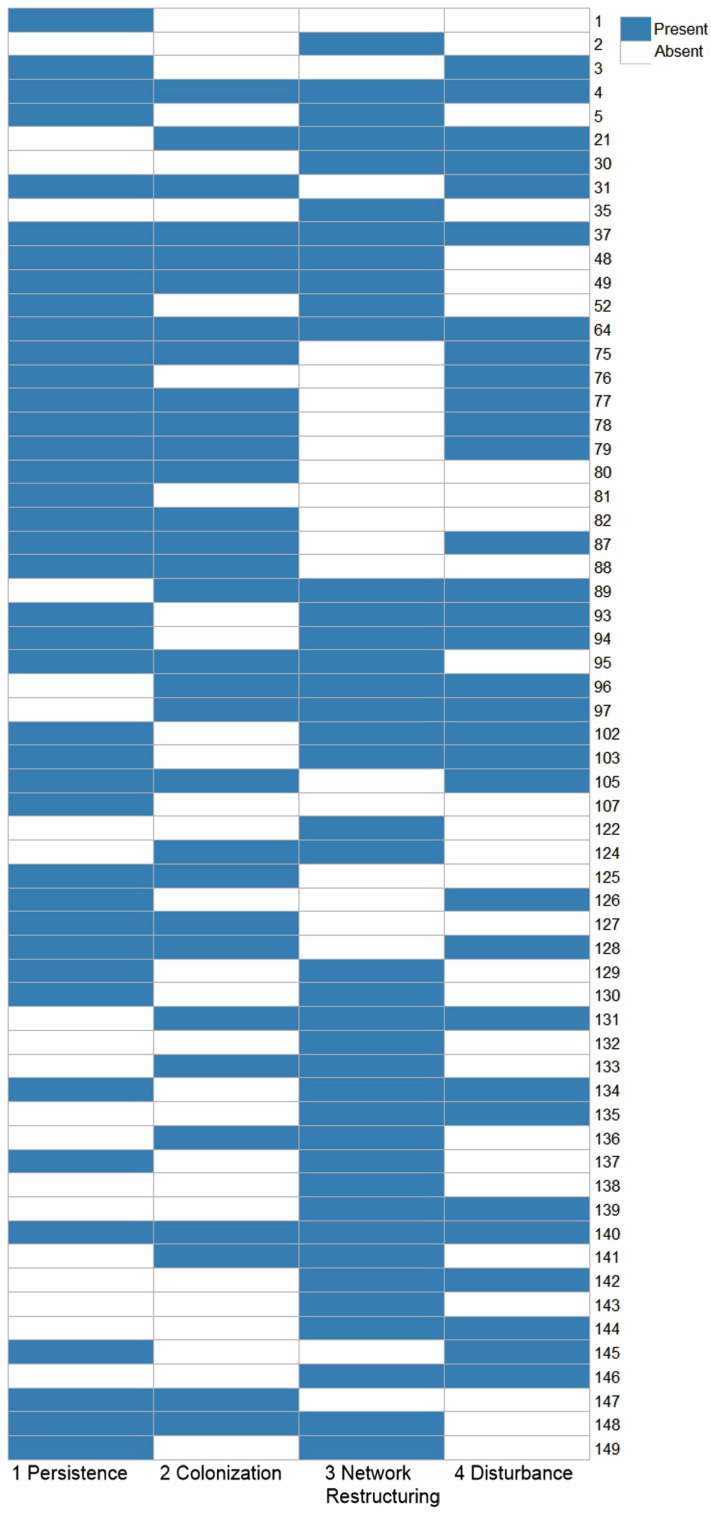
Distribution of studies across mechanistic pathways linking border vegetation to pest dynamics. Heatmap showing the classification of empirical and conceptual studies according to four mechanistic pathways: (1 Persistence) host reservoirs and selective filters, (2 Colonization) modification of pest behavior and colonization, (3 Network Restructuring) restructuring of trophic and disease dynamics, and (4 Disturbance) management as a selective force. Each row represents a study (numbered as their citation), and each column a mechanism; colored cells indicate that a study was assigned to a given mechanism (blue = present, white = absent). Studies may be associated with multiple mechanisms. Classification was based on explicit evidence or interpretation of how border vegetation influenced pest persistence, movement, multitrophic interactions, or responses to disturbance, following the framework developed in this review. The full list of studies and detailed classification is provided in Table S1 (see Supplemental Material—Methods S2 for literature review details).

Borders are therefore commonly portrayed as beneficial habitat strips that enhance biodiversity and biological control (e.g., [33,34,35] and Figure 2), where this study summarizes a structured, synthesis of representative empirical and conceptual studies based on their relevance to field border vegetation and pest-associated ecological processes and their interpretability within the mechanistic framework developed in this review (Figure 2). Yet border vegetation is dominated by weeds, many of which are closely related to crops or share herbivores, pathogens, and soil biota with them [36,37,38]. This functional and phylogenetic similarity creates ecological continuity between border plant communities and crops, meaning that borders may act not only as reservoirs for beneficial organisms, but also as habitats that sustain pest populations, enable their survival between cropping periods, and influence their movement into fields.

Resource-based coexistence theory further suggests that plant diversity reflects heterogeneity in resource supply and environmental filters [39,40,41,42,43]. Where resource pools are diverse and spatially variable, weed communities may include species with a wide range of functional traits, potentially reducing dominance by highly competitive species (e.g., Figure 2 and Table S1). In contrast, simplified or strongly filtered systems—such as margins exposed to frequent mowing, herbicide drift, or nutrient enrichment—can favor species whose traits align closely with crop resource-use strategies, intensifying competition and ecological similarity between weeds and crops (e.g., Figure 2 and Table S1). Phylogenetic and functional similarity between weeds and crops often predicts overlap in herbivore and pathogen assemblages, because closely related plants tend to share secondary metabolites, structural defenses, and phenological schedules that shape enemy host-use patterns [44,45,46,47]. Thus, environmental filtering that selects weed species with crop-like resource-use traits may simultaneously select plants that are suitable hosts for crop-associated pests. The same processes that structure weed diversity through resource heterogeneity and environmental filtering may therefore determine pest host availability, spillover risk, and transmission pathways.

From this perspective, field borders represent more than peripheral habitats; they are dynamic ecological assemblages shaped by disturbance regimes, management filters, and dispersal processes whose composition influences not only plant competition but also the persistence and movement of associated consumer and pathogen communities (see Figure 2). For example, brassicaceous weeds in borders can sustain aphids and flea beetles that exploit oilseed crops, while solanaceous weeds such as *Solanum nigrum* host herbivores and pathogens of tomato and potato [48,49]. Borders can additionally provide overwintering refuge for hemipterans, coleopterans, and lepidopterans, and maintain perennial or soil-borne pathogen reservoirs, linking pest dynamics across seasons (Table S1). In metacommunity terms, pest populations occupy spatially structured host patches connected by dispersal, with local dynamics shaped by colonization–extinction tradeoffs and species sorting across environmental gradients [50,51]. Border vegetation modifies both patch quality (host suitability, enemy pressure, microclimate) and connectivity (structural permeability, spatial proximity), thereby altering regional pest persistence [52]. Borders can therefore function as sources (high host quality, low enemy suppression), sinks (high mortality due to enemies or unsuitable hosts), or ecological filters that selectively facilitate or impede movement depending on vegetation structure, chemistry, and phenology.

Despite these dynamics, plant pest ecology and integrated pest management (IPM) have only partially incorporated border plant communities into conceptual or applied frameworks [6,28,29,53]. IPM has traditionally emphasized in-field practices and landscape composition at coarse spatial scales, while borders are often treated as static margin features categorized simply as “present versus absent” or “flower strip versus unmanaged” [54]. This reductionist view obscures how border plant community structure—especially diversity, dominance patterns, and functional similarity to crops—regulates pest dynamics through bottom-up pathways. Dominance by a few highly suitable host weeds may concentrate pest populations, whereas more diverse assemblages containing non-host or chemically distinct species may dilute host signals, alter pest behavior, and disrupt performance. Because weed communities are shaped by succession, disturbance, dispersal, and management inputs, borders act as dynamic ecological nodes whose role in pest systems can shift across seasons and years.

From a community and metacommunity ecology perspective, border vegetation can influence pest populations through multiple interacting mechanisms that generate testable predictions and remain underexplored in agroecology (Figure 1). We summarize these processes into four hypotheses. First—*persistence hypothesis*—proposes that seasonal host continuity in borders, including perennial or off-season host plants, reduces pest extinction risk and enhances early-season colonization in adjacent crops [31,55,56]. Second—*host similarity hypothesis*—predicts that border plant composition regulates pest abundance through resource concentration when crop-like hosts dominate, or resource dilution [52,57] when taxonomic and functional diversity increases [21,58]. Third—*network restructuring hypothesis*—suggests that border vegetation modifies multitrophic interactions by simultaneously supporting pests, natural enemies, and pathogens, generating context-dependent and potentially nonlinear effects on pest suppression [59,60,61]. Fourth—*disturbance filtering hypothesis*—proposes that management practices such as mowing and herbicide drift act as environmental filters shaping plant community structure and indirectly influencing pest movement, survival, and spillover [31,62]. Together, these hypotheses (Figure 1) highlight that field borders regulate pest dynamics not simply through species richness, but through interacting processes of persistence, resource use, trophic structure, and disturbance regimes.

Management practices further modulate these pathways by altering successional trajectories and environmental filters [32,39,42]. Mowing regimes, nutrient runoff, tillage intensity near borders, and intentional seeding of floral mixes influence border plant diversity, phenology, and structural complexity [26,28,32]. Frequent disturbance may maintain communities dominated by ruderal annuals closely aligned with crop pest life cycles, while reduced disturbance can allow the development of perennial vegetation that modifies wind flow, humidity, and thermal buffering, with consequences for insect behavior and pathogen persistence. Consequently, the same border can shift roles over time—acting as a pest refuge under one management regime and as a behavioral barrier, dilution system, or enemy-supporting habitat under another (Figure 1). Current frameworks lack a predictive structure linking border plant community assembly processes to pest outcomes, leaving managers without guidance on how to design borders that suppress pest carryover while maintaining pollination and natural enemy services.

Here, we propose that field borders also function as ecological gatekeepers that mediate pest movement, persistence, and interaction networks between surrounding landscapes and crops. This framing shift borders from static habitat features to dynamic components of agroecosystem metacommunities whose effects emerge from interactions among dispersal, environmental filtering, and multitrophic interactions across scales (Figure 1). We synthesize theory and empirical evidence (Figure 2) to argue that border plant diversity and composition determine whether borders act as pest sources, sinks, or ecological filters. We outline four mechanistic pathways (Figure 1): (1) border weeds regulate pest persistence through alternative hosts and overwintering habitat, with diversity influencing resource concentration versus dilution; (2) border vegetation shapes pest colonization by modifying visual structure and volatile-mediated host location; (3) border plant communities restructure multitrophic interactions by simultaneously supporting enemies, pests, and pathogens; and (4) management drives border diversity and disturbance regimes, thereby modulating the strength and direction of these pathways. By reframing weeds as both plant pests and facilitators of pest metacommunity dynamics, this framework integrates weed ecology, coexistence theory, movement ecology, multitrophic interactions, and IPM, and generates testable predictions for designing border communities that reduce pest carryover while maintaining other ecosystem services.

## 2. Empirical Evidence from Crop Border Weed Surveys

### 2.1. Using Border Surveys to Test the Framework

Previous studies of crop borders and edges (Table S1) have largely emphasized weed biodiversity as a mechanism to enhance natural enemies and pollinators, often treating pests as secondary outcomes within biological control frameworks. While those works demonstrated the conservation value of vegetated margins, it rarely evaluates the mechanistic pathways that also links border plant composition to pest persistence, colonization, or spillover into crop interiors (see [52]). In particular, few studies account for the fact that border weeds may simultaneously provide overwintering habitat, alternative hosts, or pathogen reservoirs that increase pest pressure when crops are absent [52].

To ground our conceptual framework in empirical patterns (Figure 2), we conducted vegetation surveys focused not only on pollinators and beneficial insects but mainly on pest-plant associations across foliar and root-feeding guilds. Borders were defined as vegetated strips directly adjacent to cultivated fields, extending more than 2 m from the crop edge (to minimize immediate crop-edge effects; see Box 1). These strips usually were subject to lower disturbance than the crop interior but experienced periodic management such as mowing, herbicide drift, or edge tillage. Generally (of all the ten farms we have been surveying since 2023), borders are kept in ranges from 5 to 10 m in width and 10 to 30 m in length (see Supplemental Material—Methods S1). Operationally, they represent the interface between intensively managed crop fields and semi-managed or ruderal habitats. Weed communities were sampled using quadrats placed systematically along the crop-border interface (see method at [44]; see Supplemental Material—Methods S1). Sampling was conducted throughout the growing seasons to capture the vegetation structure coinciding with pest colonization and crop development. For each quadrat, plant species presence, relative abundance, and growth form were recorded (see Supplemental Material).

Box 1 **Edge vs. Border.**

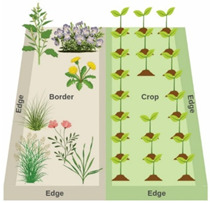

**Edge (Ecotone):** An edge is a transitional zone where two habitat types meet (e.g., crop–forest, crop–grassland). It is characterized by gradients in environmental conditions and species composition, often leading to changes in species interactions and movement. **Border:** A border is the vegetated strip adjacent to a field or habitat boundary. Borders may be unmanaged, semi-managed, or intentionally planted, and their plant composition and structure vary depending on management. **Key distinction:** Edges are ecological transitions; borders are the plant communities that often create, shape, or modify those transitions. [Figure created with the help of BioRender.com]

Simultaneously, all insects but especially pest populations (including foliar insects and soil-associated arthropods) were monitored across the growing season, with particular attention to host use and temporal variation in abundance. This design does not aim to provide exhaustive floristic inventories. Rather, it characterizes the structural and functional attributes of border weed communities that are most likely to influence pest persistence, movement, and multitrophic interaction dynamics within adjacent crops.

### 2.2. Empirical Signals Supporting the Proposed Mechanisms

Several consistent patterns emerging from these surveys align with the four mechanistic pathways from the literature search (Figure 2; see Supplemental Material—Methods S2) developed in Section 3.

**Figure 3:**
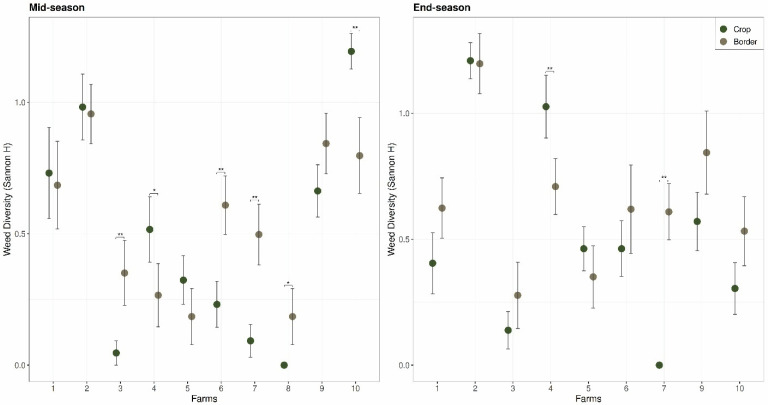
Crop and border weed community diversity across farms. Shannon diversity (H′; mean ± SE) of weed communities in crop interiors (green) and adjacent field borders (brown) across ten farms in northern Utah. Panels show mid-season (**left**) and end-season (**right**) surveys. Differences between crop and border habitats reflect variation in disturbance intensity and management regimes across the agricultural gradient. Lower diversity values indicate stronger dominance by a small number of taxa, whereas higher values reflect greater community evenness. Asterisks indicate significant differences between crop and border habitats (* *p* < 0.05, ** *p* < 0.001).

**Figure 4:**
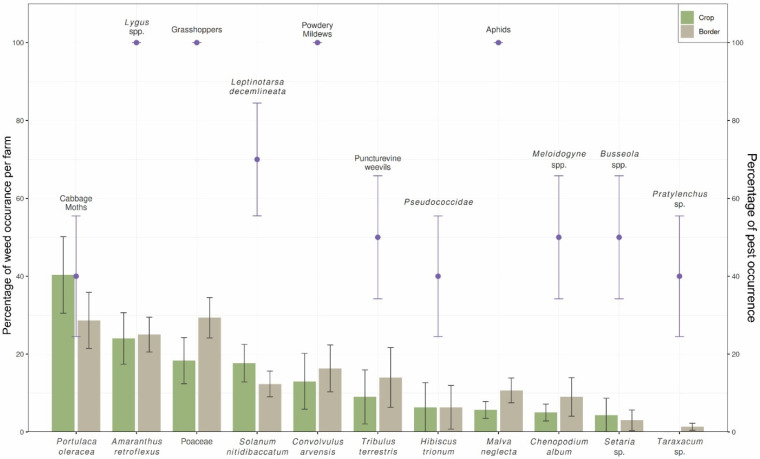
Associations between dominant border and crop weed taxa and pest occurrence across farms. Relative abundance of dominant weed taxa (bar plots; mean ± SE) and associated pest occurrence (purple points; mean ± SE) recorded in crop interiors and adjacent field borders. Weed taxa include those most frequently observed across surveys, and pest groups include foliar herbivores, pathogen vectors, plant pathogens, and soil-associated pests. No significant differences were observed in the relative abundance of weed taxa between crop and border habitats, nor in associated pest occurrence linked to these taxa. Accordingly, this figure is intended to illustrate ecological associations between weed identity and pest presence rather than statistical differences between habitat types. Although associations are not exclusive, patterns suggest that a subset of dominant weed taxa consistently co-occurs with key pest guilds, highlighting the importance of plant identity and dominance in shaping multitrophic interactions across crop–border interfaces.

Across farms, weed communities exhibited pronounced dominance structure, with a limited subset of species contributing disproportionately to total cover (Figure 3). In several borders, dominant taxa included species with documented associations to crop pests (Figure 4; Table S1). This pattern is particularly important in the context of host-reservoir dynamics (*persistence hypothesis*). Where dominant weeds are competent alternative hosts or overwintering substrates, even moderate overall diversity may mask high functional host availability (Figure 3). Moreover, the coexistence of annual and perennial species indicates temporal resource continuity (Figure 4). Perennial and winter-active species provide vegetative structure beyond peak crop growth, while summer annuals overlap with crop phenology. Such life-history complementarity creates the conditions predicted under the persistence hypothesis: reduced seasonal bottlenecks and increased probability of early-season pest colonization in adjacent crops. Thus, our survey suggests that pest risk is more closely associated with dominance by functionally suitable hosts than with total plant richness per se.

Structural and compositional variation among borders (Figure 3) indicates that pests encounter different sensory and architectural environments at the crop-border interface. Borders dominated by crop-related taxa (e.g., solanaceous or brassicaceous weeds) create strong phylogenetic and chemical similarity to crop hosts (*host similarity hypothesis*). Such similarity likely reinforces visual and olfactory continuity across the boundary, facilitating directed movement into fields (Figure 4). In contrast, borders with higher representation of grasses and morphologically distinct forbs generate greater structural heterogeneity. Variation in plant height, leaf morphology, and growth form increases visual complexity and may dilute host-specific volatile cues. These differences imply that borders may function either as behavioral conduits or as sensory filters, depending on community composition. Additionally, phenological overlap between dominant weeds and crops suggests potential synchrony in host availability. Where dominant border species share seasonal timing with crops, colonization pressure may intensify; where phenologies are asynchronous, movement into crops may be delayed or reduced.

The pest assemblages recorded in association with border weeds (Figure 4) included both foliar herbivores and belowground organisms, indicating that border vegetation participates in multitrophic interactions spanning above- and belowground systems. Several weed taxa were associated with multiple pest groups, including herbivores, vectors, and plant-parasitic nematodes (Figure 4), suggesting that single plant species can link multiple trophic pathways (*network restructuring hypothesis*). Importantly, pest occurrence was not uniform across weed taxa. Instead, specific weeds supported disproportionately high pest presence, and many other species besides the dominant ones shown on Figure 4. This uneven association structure indicates that borders contribute to interaction rewiring through species identity rather than richness alone. Furthermore, the simultaneous presence of herbivores and documented pathogen hosts implies that borders may restructure both predator-prey and disease transmission networks. The co-occurrence of vector-associated weeds and foliar pathogens supports the hypothesis that borders influence both trophic and epidemiological dynamics extending into crops.

Variation in weed richness and composition between crop interiors and borders (Figure 3) reflects differential disturbance regimes (*disturbance filtering hypothesis*). Crop interiors, subject to tillage and herbicide application, exhibited reduced structural diversity relative to borders. Borders, though periodically managed, supported a broader mixture of functional groups, including perennial species absent from the crop interior. Among farms, variability in dominance structure suggests differences in management history or disturbance frequency. Borders characterized by strong dominance of ruderal annuals likely reflect frequent disturbance, whereas borders with greater structural heterogeneity and perennial presence may represent lower disturbance intensity. These compositional differences correspond to predicted shifts in pest pathways: simplified, annual-dominated borders may promote host continuity aligned with crop phenology, while perennial-dominated borders may enhance overwinter survival and microclimatic buffering.

Collectively, these patterns found in our long term survey indicate that crop borders are structured ecological communities with predictable dominance patterns, functional differentiation, and documented pest associations. They are not neutral biodiversity reservoirs but potential regulators of pest persistence, colonization, multitrophic interactions, and disease dynamics. Although temporally limited, this proof-of-concept survey provides empirical grounding for the four mechanistic pathways developed in Section 3. The following section generalizes these patterns into a unified conceptual framework linking border composition, disturbance history, and pest dynamics across agroecosystems.

## 3. Mechanistic Pathways Linking Border Weeds to Plant Pest Ecology

Efforts to manage agricultural weeds have increasingly moved beyond simple eradication toward approaches that incorporate biodiversity, ecological processes, and long-term system resilience. Diversified crop rotations, perennial crop systems, cover cropping, and reduced tillage are now promoted as strategies that improve soil structure, nutrient cycling, and biological regulation [28,29,63]. These shifts in management have not only altered in-field weed communities but have also reshaped vegetation dynamics in field borders. Reduced disturbance intensity altered nutrient flows, and changes in crop phenology can favor species typical of ruderal, meadow, or semi-natural habitats [49,64]. As a result, borders of farms ranges from bare soil to compositionally complex vegetation, with increased representation of perennial species, structurally diverse growth forms, and taxa with traits associated with persistence and dispersal (Box 1; [65,66]). However, greater plant diversity does not automatically translate into beneficial outcomes for pest (e.g., insects, nematodes) regulation. Ecological theory emphasizes that biodiversity effects depend on plant species identity, functional traits, and interaction networks rather than richness alone [67,68,69]. In border habitats, increased diversity may include species that are closely related to crops, share herbivores or pathogens, or possess traits that enhance pest survival and dispersal [70,71]. Thus, diversification of border vegetation can simultaneously enhance ecosystem services (e.g., pollination, natural enemy habitat) and strengthen ecological pathways that facilitate pest persistence. This duality reflects the broader ecological principle that communities function as networks of interacting species, where positive and negative effects emerge from the same structural complexity.

Weed management strategies such as cover cropping, reduced herbicide inputs, and conservation tillage are often implemented within fields to suppress weeds through competition, resource capture, and soil-mediated processes, including plant–soil feedbacks [57,72,73,74]. Yet these approaches frequently overlook the vegetation at field borders, where disturbance regimes, management filters, and dispersal processes differ markedly from the crop interior. Borders receive herbicide drift, episodic mowing, soil movement, and propagule inputs from surrounding habitats, creating conditions that promote dynamic, successional weed assemblages. Because these assemblages persist at the crop interface, they influence the movement of organisms between semi-natural habitats and crops [52,72]. Understanding weed dynamics without considering borders therefore provides only a partial picture of agroecosystem functioning. Moreover, weeds are not only competitors of crops but also participants in multitrophic interactions that include herbivores, pathogens, predators, parasitoids, and soil biota. Border weeds can act as alternative hosts, refuges, or resource nodes within these interaction networks [52,74]. Their phenology, architecture, chemistry, and spatial arrangement influence how pests locate hosts, survive unfavorable periods, and interact with natural enemies [72,73,74]. These processes operate across seasons and spatial scales, linking local population dynamics with landscape-level movement. In this sense, border weed communities function as ecological interfaces that regulate energy flow, organism movement, and interaction strengths between habitat patches (Figure 1).

**Figure 5:**
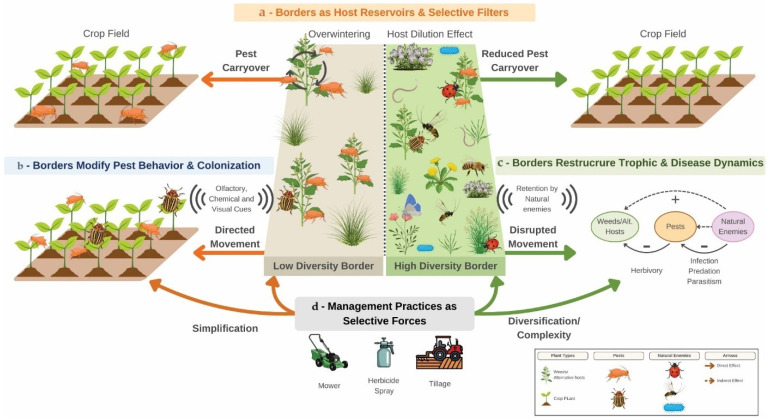
Conceptual framework of field borders as ecological gatekeepers in agricultural pest metacommunities. The diagram illustrates how border vegetation regulates pest persistence, movement, and multitrophic interactions through four interconnected mechanisms. Borders are dynamic plant assemblages that vary in composition and diversity, shaping whether they function as pest sources, sinks, or ecological filters. (**a**) Host reservoirs and selective filters: Border weeds provide alternative hosts and overwintering habitat that support pest persistence between cropping seasons. (**b**) Modification of pest behavior and colonization: Vegetation structure and chemical cues influence pest movement and host-finding at field edges. (**c**) Restructuring of trophic and disease dynamics: Border plant communities shape interactions among pests, natural enemies, alternative hosts, and pathogens. (**d**) Management as a selective force: Disturbance and management practices filter plant communities and indirectly regulate pest-associated interactions. Arrows indicate movement of pests, natural enemies, and pathogens between borders and crops. The framework emphasizes that border plant composition and diversity regulate pest metacommunity dynamics across agricultural landscapes. [Figure created using BioRender.com].

Field borders are more than static edge habitats (Box 1); they are dynamic ecological assemblages shaped by disturbance regimes, management filters, and dispersal processes. Borders act as nodes in pest metacommunities, connecting weed, pest, and beneficial populations across landscapes ([52]; Figure 2 and Table S1). To move beyond descriptive accounts of border biodiversity, it is therefore necessary to identify the ecological mechanisms through which border plant communities influence pest dynamics (Figure 1). While most studies focus on their value for pollinators and natural enemies, we argue that border plant communities also play critical roles in shaping pest persistence, colonization, and multitrophic interactions (Figure 2). Border weeds affect plant pest ecology through predictable pathways grounded in community ecology, movement ecology, and multitrophic theory [52,74]. Using a combination of empirical surveys and literature synthesis (Figure 2), we propose four interrelated mechanistic pathways that link border vegetation structure and composition to pest persistence, colonization, interaction networks, and management outcomes (Figure 5 and Table 1).

### 3.1. Borders as Host Reservoirs and Selective Filters

A central principle from metapopulation and metacommunity theory is that species persistence in patchy environments depends on the balance between local extinction and recolonization (Figure 5a and Table 1). In agricultural landscapes, crop fields represent temporally unstable habitat patches: they are harvested, rotated, or treated, periodically removing host resources. Under such conditions, pest populations often persist only if alternative habitat patches provide refuge, resources, or overwintering sites during periods when crops are absent or unsuitable. Field borders, which maintain semi-permanent vegetation, can therefore function as habitat reservoirs that buffer pest populations against seasonal bottlenecks (i.e., [52,74]). From this perspective, border weeds contribute to temporal resource continuity (Figure 5a). Perennial or phenologically asynchronous plant species maintain living tissue, root systems, litter layers, and microhabitats that allow herbivorous insects and plant pathogens to survive between crop cycles. Overwintering stages of hemipterans, coleopterans, and lepidopterans frequently occur in non-crop vegetation characteristic of field margins (Table S1). Empirical evidence supports this mechanism (Figure 2). Stink bugs and carrot weevils overwinter in weedy borders and exhibit higher early-season densities near field edges [75,76]. Thrips and russet mites utilize weeds as “green bridges” between crop cycles, with genetic and demographic evidence confirming spillover into crops [77,78,79]. Crucifer flea beetles overwinter in vegetation surrounding *Brassica* fields [80], and plant viruses such as alfalfa mosaic virus and cucumber mosaic virus persist in weed hosts and are transmitted to crops by aphids [81]. Similarly, plant pathogens with broad host ranges may persist in asymptomatic weed hosts or in soil associated with compatible plant species. Collectively, these studies illustrate a metapopulation rescue effect in which border habitats reduce local extinction risk of pests and increase early-season colonization probability—a classic metapopulation rescue effect.

However, borders do not function uniformly as pest reservoirs; their role depends on plant community composition and diversity. Here, borders also act as selective ecological filters that determine which host species—and therefore which pest species—are maintained (Figure 5a; Table 1). In low-diversity borders dominated by crop congeners or highly suitable alternative hosts, several processes converge to elevate pest carryover risk (i.e., [52]). Dominance by functionally or phylogenetically similar species increases host continuity across seasons. Shared traits between weeds and crops may also promote similar soil microbial assemblages and plant–soil feedbacks that favor pests or pathogens adapted to those host lineages (Table 1). Moreover, concentrated host availability can allow pest populations to build locally before spilling over into adjacent crops when conditions become favorable [52,74]. In contrast, higher-diversity borders may modify these dynamics through mechanisms analogous to resource dilution and associational resistance (Figure 5a; Table 1). When plant communities contain a broader range of taxonomic identities and chemical traits, suitable hosts for a given pest may be spatially diluted among non-host species. Increased heterogeneity can interfere with host-finding, reduce per-host colonization rates, and limit population growth of specialist pests [52,74]. Importantly, diversity alone does not eliminate risk. Even diverse borders may function as reservoirs if key host species are abundant, perennial, or phenologically well aligned with pest life cycles. Thus, plant identity and dominance structure can outweigh richness per se (Table 1). Conversely, some weeds may act as ecological sinks, receiving oviposition but failing to support pest development [82].

Our survey results reinforce this distinction. Although species richness and functional diversity varied among borders, pest-relevant risk was not consistently associated with higher or lower diversity per se (Figure 3). Instead, risk was linked to dominance structure patterns: a small number of species often accounted for a disproportionate share of vegetative cover and therefore host availability. Where dominant taxa were documented alternative hosts or overwintering resources for crop pests, borders were more likely to function as reservoirs, regardless of total richness (Figure 4). Where dominant species were non-hosts or structurally unsuitable, borders were more likely to act as ecological filters that limited pest persistence (Figure 5a). Thus, border plant diversity alone is an insufficient predictor of pest risk. Rather, risk emerges when low-diversity borders are dominated by plant species that serve as effective alternative or overwintering hosts, while diverse borders can still pose risk if key host species achieve high abundance. Understanding borders as host reservoirs and selective filters therefore requires integrating diversity metrics with species identity, functional traits, and dominance patterns.

### 3.2. Borders Modify Pest Behavior and Colonization Dynamics

Beyond sustaining pest populations across seasons, border vegetation also shapes how pests move through landscapes and locate crop hosts (Figure 5b; Table 1). Colonization dynamics in herbivorous insects are strongly governed by sensory ecology: individuals rely on visual contrasts, plant architecture, and volatile chemical cues to detect, orient toward, and accept host plants [71,83,84,85]. In heterogeneous environments, the reliability and clarity of these signals determine search efficiency, habitat selection, and ultimately colonization rates. Borders therefore function not only as reservoirs of organisms, but as behavioral landscapes that mediate the transition of pests from non-crop habitats into crop fields ([52]; Figure 2). Theories of resource concentration and associational resistance provide a useful framework for understanding these dynamics (Figure 2). When host plants are aggregated and sensory signals are strong and predictable, herbivores more easily locate and remain on suitable hosts [71,84,86]. Conversely, when hosts are embedded within taxonomically and structurally diverse vegetation, mixed sensory cues may disrupt orientation, reduce host-finding efficiency, or alter movement trajectories [21,22,74]. In border habitats, plant community composition determines whether pests experience a simplified, cue-rich environment that guides them toward crops or a heterogeneous environment that interferes with directed colonization (Figure 5b).

In low-diversity borders dominated by plant species that are taxonomically or chemically similar to crops, visual and olfactory cues may form strong directional gradients toward adjacent fields (Figure 5b; Table 1; [44,45,47]). Uniform plant architecture and concentrated host volatiles can facilitate directed movement into crops, particularly when border hosts are phenologically synchronized with crop development. Empirical evidence supports this host similarity hypothesis. In Brassica systems, the presence of wild congeners in field margins increases flea beetle damage in crops [48,49], consistent with associational susceptibility driven by shared host cues. Similarly, thrips invasion intensity correlates with weed abundance and phenological overlap, indicating that seasonal synchrony between border vegetation and crops regulates colonization pressure [78]. Border-mediated colonization gradients further demonstrate behavioral filtering: cotton fleahopper densities decline from field borders inward when adjacent vegetation is present [87], and Colorado potato beetles aggregate at field borders where vegetation structure influences overwintering distribution and subsequent movement into crops [88]. Together, these studies indicate that host-dominated borders can function as behavioral conduits that amplify colonization pressure (see Table S1).

In contrast, high-diversity borders generate heterogeneous visual and chemical environments. Variation in plant height, leaf morphology, growth form, and spectral reflectance alters the visual background against which crops are detected, while mixtures of plant volatiles may mask or dilute host-specific chemical cues (Figure 5b; Table 1). This sensory “noise” can reduce the efficiency of host location, increase exploratory movement, or prolong pest residence within border vegetation rather than promoting directed entry into crops. Asynchronous phenologies among border species may further decouple pest emergence from optimal crop stages, modifying both timing and intensity of colonization. Studies showing reduced herbivory in more floristically diverse margins [49,89] are consistent with associational resistance and resource dilution mechanisms. Structural diversity, reflected in variation in growth forms (prostrate forbs, upright herbs, grasses), suggests that borders differ in architectural complexity, flight behavior, and landing probability. Phenological overlap between dominant border species and crops (Figure 3) indicates whether borders provide temporally aligned cues that could facilitate pest transfer. The relative representation of crop-related taxa determines similarity of visual and chemical signals at the crop-border interface (Figure 5b; Table 1). Thus, pest colonization is jointly shaped by taxonomic similarity, structural configuration, and phenological alignment between border vegetation and crops. Therefore, suggesting that borders can either amplify or disrupt host-location processes, depending on community composition and structure (Figure 5b).

Overall, borders influence pest colonization not merely by harboring individuals, but by structuring the sensory environment through which pests perceive and navigate the landscape (Figure 5b; Table 1). Pest abundance and early-season pressure tend to increase when borders are dominated by crop-like or phenologically synchronized hosts, creating concentrated and reliable cues. In contrast, more taxonomically and structurally diverse borders may disrupt host-location processes, reduce colonization efficiency, or retain pests within border habitats. Understanding borders as behavioral filters highlights that colonization risk emerges from plant identity, dominance, and community structure—not from diversity levels alone.

### 3.3. Borders Restructure Trophic and Disease Dynamics

Ecological communities are structured not only by species identities, but by the network of interactions among them (Figure 5c; Table 1). Food web theory emphasizes that changes in community composition can rewire interaction networks, altering energy flow, interaction strengths, and the balance between top-down and bottom-up control [90,91,92]. In agroecosystems, field borders represent semi-permanent habitat patches where plants, herbivores, natural enemies, pathogens, and mutualists co-occur. Because these organisms are connected through trophic and disease transmission pathways, border plant communities can reorganize multitrophic networks that extend into adjacent crops. Border weeds simultaneously support multiple trophic levels (Figure 2 and Figure 4; [52]). Weed species provide foliage and roots for herbivores, nectar and pollen for predators and parasitoids, shelter for overwintering arthropods, and living tissue or microhabitats for fungal, bacterial, and viral pathogens (Figure 5c; Table 1). As a result, borders often increase network complexity. However, greater complexity does not inherently translate into stronger pest suppression. The outcome depends on how plant diversity reshapes the abundance, timing, and connectivity of interacting species.

In high-diversity borders, a broader range of plant species can sustain more diverse assemblages of predators and parasitoids by supplying nectar, alternative prey, and refuge. Empirical studies show that perennial margins support overwintering ground-dwelling predators and are associated with 15–65% lower aphid infestations in some systems [93]. Margins can act as overwintering refuges for aphid predators [94], and parasitoids may persist on weed-hosted aphid populations before moving into crops [95]. Flower-rich margins frequently increase predator abundance and biological control potential [89,96]. These findings (see Table S1) suggest that increased plant diversity can enhance functional complementarity among natural enemies and strengthen top-down regulation. Yet trophic restructuring is not uniformly suppressive. The same diverse borders that support natural enemies may also maintain multiple pest species and pathogen reservoirs (Figure 2 and Figure 4). Even moderate diverse weed communities (Figure 3) can host asymptomatic pathogen carriers, sustain vector populations such as aphids, leafhoppers, or thrips, and provide sequential feeding opportunities that connect weeds to crops (Figure 2). Wild Brassicaceae in margins, for example, have been shown to increase pest damage despite simultaneously supporting natural enemies [48,49]. Seed banks may increase under flower strips [3], and predator spillover into crops is sometimes weak or context-dependent [97]. Increased network complexity can also introduce indirect interactions such as intraguild predation, hyperparasitism, or phenological mismatches between enemies and pest peaks, potentially weakening effective control. Thus, diversity can simultaneously strengthen and dilute top-down control (Figure 5c and Table 1).

Low-diversity borders produce different, but not necessarily safer, network configurations. Dominance by one or a few host weeds can promote specialist pest populations tightly linked to those hosts (Figure 4). Reduced plant and structural diversity may limit natural enemy abundance and functional redundancy, weakening top-down control (Table 1). At the same time, simplified vegetation can increase vector efficiency by concentrating feeding sites and creating unobstructed pathways between infected weeds and crops, potentially increasing disease transmission rates. In such cases, interaction networks are simpler but may be more strongly biased toward pest persistence and pathogen spread.

Importantly, plant pests and pathogens must be considered explicitly within these networks (Figure 5c). Weeds can serve as reservoirs for soil-borne pathogens and nematodes associated with roots, as well as foliar viruses and bacteria transmitted by insect vectors (Figure 2 and Table 1). Many herbivorous insects overwinter or complete early life stages on non-crop plants before shifting to crops, creating sequential feeding pathways that link border vegetation to crop infection or damage (Table 1; [75,77,80,98]). These linkages connect above- and belowground interactions, integrating trophic and disease dynamics across spatial boundaries (Figure 5c).

Overall, border vegetation restructures multitrophic networks rather than simply adding beneficial species. Plant diversity mediates trade-offs between biological control and pest or pathogen persistence, and the net outcome depends on dominance structure, host identity, phenological alignment, and interaction strength within the network. Borders should therefore be evaluated as multitrophic systems in which plants simultaneously support enemies, pests, vectors, and disease agents (Table 1). Understanding how border composition rewires interaction networks is essential for predicting when increased diversity enhances pest suppression versus when it stabilizes or amplifies pest and pathogen populations.

### 3.4. Management Practices as Selective Forces

Field borders exist at the interface of ecological processes and human decision-making, and their plant communities are shaped by repeated disturbance, selective removal, and occasional intentional planting (Figure 5d; Table 1). Disturbance ecology and environmental filtering theory provide a useful framework: disturbances act as selective pressures that favor species with traits adapted to specific regimes of stress, resource pulses, and competitive release [99,100]. In agroecosystems, management practices—mowing frequency, herbicide drift, tillage intensity, grazing, or the establishment of flower strips—function as recurrent filters that determine which plant functional groups persist along field borders. In turn, this selective filtering governs which pests, pathogens, and natural enemies are supported.

From a metacommunity perspective, border management creates local filters that shape species sorting at the landscape scale (Figure 5d; Table 1). Borders are not passive vegetation remnants; they are selectively structured nodes within a network of habitat patches that mediate colonization, extinction, and interaction pathways. Just as dispersal limitation and local extinction structure metacommunities, management-induced filtering determines which weeds persist and which associated organisms disperse into adjacent crops (Figure 2). Border diversity is therefore not incidental—it reflects the cumulative outcome of repeated management decisions (Figure 5d; Table 1).

Over-managed borders—subjected to frequent mowing, herbicide exposure, or aggressive tillage—tend to harbor simplified communities dominated by disturbance-tolerant annuals and ruderal species. These plants often share life-history traits, phenology, and sometimes phylogenetic affinity with crop species, creating strong host continuity for specialist pests (Table S1). Reduced richness and evenness increase the likelihood that a small number of highly suitable host plants dominate, concentrating pest populations and facilitating overwintering or early-season colonization. Empirical work indicates that mowing and herbicide regimes can substantially alter weed composition without necessarily reducing pest pressure [76]. Simplified border habitats may therefore reduce structural complexity while maintaining or even intensifying pest pathways (Figure 5d; Table 1).

Conversely, under-managed borders—where disturbance is minimal—may transition toward perennial dominance and greater structural complexity [7,23,66,101]. Such borders often support more diverse arthropod communities, including predators, parasitoids, and pollinators. Disturbance regimes influence successional stage, vegetation architecture, and microclimatic stability, all of which affect both pests and natural enemies [102,103]. However, reduced disturbance can also allow persistent pest refugees to develop. Perennial vegetation may stabilize microhabitats across cropping cycles, supporting overwintering stages and long-term pathogen reservoirs. Low-tillage systems and flower strips, for example, can increase weed seed banks near field borders [3], potentially expanding the pool of alternative hosts (Figure 5d; Table 1).

Border structure and cultivation practices further influence pest aggregation and dispersal patterns (e.g., [104]). Border-associated pests such as the wheat stem sawfly show strong aggregation responses to vegetation structure and border configuration [105], illustrating how management decisions can indirectly shape colonization dynamics (Figure 2). In this way, disturbance regimes alter not only plant diversity but also the spatial configuration of pest pathways and trophic interactions. Importantly, both extremes—over-management and neglect—can increase pest risk, albeit through different mechanisms. Intensive disturbance may simplify communities and concentrate crop-like hosts, enhancing resource continuity and colonization efficiency. Minimal disturbance may promote perennial dominance and habitat continuity, increasing the persistence of pests and pathogens across seasons. The relationship between management and pest outcomes is therefore nonlinear and mediated by species identity, functional traits, and dominance patterns rather than richness alone [28,29]. Our data supports this filtering framework. Borders with recent mowing histories or high herbicide exposure exhibited lower species richness and evenness, with dominance by a few disturbance-adapted or crop-related weeds (Figure 3). In contrast, minimally managed borders supported taller perennials and a broader mix of grasses and forbs but also hosted pest species absent from intensively managed borders. These patterns reveal a mismatch between management intentions and ecological outcomes: efforts to simplify borders for operational convenience may inadvertently concentrate pest hosts, whereas neglect may allow stable pest refugia to form (Figure 5d; Table 1).

Therefore, management acts as a selective force that structures border plant communities and, through them, pest and multitrophic dynamics. Borders are selectively ignored relative to intensively managed crop interiors, yet they are not unmanaged; they reflect unintentional selection imposed by repeated disturbance regimes. By framing borders as managed ecological filters, we can better predict how changes in mowing, herbicide use, tillage, or restoration practices will cascade through plant communities to influence pest carryover, movement, and interaction networks across the agroecosystem.

## 4. Belowground Pathways and Plant–Soil Feedbacks

While the previous mechanisms (Section 3) were constructed based on the literature and therefore emphasize mostly aboveground interactions, border vegetation also exerts profound effects on belowground communities and processes, which in turn feedback to pest dynamics, crop health, and soil function. Roots of border weeds interact with diverse soil biota—including microbes, nematodes, and fungi—modifying nutrient cycling, microbial composition, and pathogen dynamics (see [52]). These interactions create a form of temporal and spatial memory in the soil, influencing pest populations and crop performance across seasons, much like aboveground refugia sustain insect pests [72]. Considering belowground pathways alongside aboveground mechanisms positions borders as multidimensional ecological filters, shaping both pest and beneficial organism dynamics.

Soil-mediated pest carryover (Figure 5a and Table 1): Border weeds can sustain soilborne pests and pathogens during crop absence (Figure 4), providing alternative hosts or habitat for nematodes, microbial pathogens, or insect larvae. For example, solanaceous weeds such as *Solanum nigrum* are known to host root-knot nematodes (*Meloidogyne* spp.) and bacterial pathogens that also attack tomato and potato [106,107]. Brassicaceous weeds influence soil microbial communities in ways that affect the survival of aboveground herbivores like flea beetles [108]. In metacommunity terms, these soil-mediated processes extend the “residency time” of pests across the landscape, linking borders, fields, and adjacent habitats. Therefore, we hypothesize that borders dominated by crop-congeneric weeds or functionally similar species enhance the persistence of belowground pests relative to more taxonomically and functionally diverse margins.

Plant–soil feedback effects (Figure 5b and Table 1): Weeds modify the soil microbiome and nutrient availability, generating plant–soil feedbacks (PSF) that can indirectly influence crop pests. Persistent pathogen accumulation in roots may increase pest pressure in subsequent crops (positive PSF), whereas diverse microbial communities associated with diverse weed assemblages can suppress pathogens or reduce herbivore performance (negative PSF) [72,73,109,110]. These feedbacks are often trait-mediated: root chemistry, exudate composition, and crop-weed phylogenetic relatedness determine whether soil biota amplify or inhibit pest populations [73,111,112]. Therefore, we hypothesized that high-diversity border communities reduce specialized soilborne pest success through dilution or antagonistic microbial interactions, whereas low-diversity, crop-like borders amplify pest feedbacks.

Integration with aboveground dynamics (Figure 5c and Table 1): Belowground interactions interact synergistically with aboveground mechanisms. For instance, soilborne pathogen stress can alter crop physiology and chemistry, affecting herbivore performance or susceptibility to foliar pathogens (e.g., [113,114]). Similarly, nematode-infested weeds may indirectly influence aboveground pest movement or enemy efficacy [115]. These cross-domain interactions illustrate that border vegetation functions as a coupled above-belowground ecological filter, where pest persistence, colonization, and trophic interactions are co-determined by both domains [52,112]. Therefore, we hypothesized that the strength and direction of aboveground pest pathways are modulated by belowground community composition, such that the same border may act as a pest refuge or filter depending on soil-mediated interactions.

Preliminary surveys and other works (Figure 2 and Table S1) indicate that several dominant border weeds in our study host soilborne pathogens and nematodes with known crop associations (Figure 4). These are aligned with published evidence of PSFs and belowground pest dynamics, supporting the notion that borders serve as multidimensional ecological nodes influencing metacommunity dynamics across spatial and temporal scales [112,116,117,118,119]. Taken together, above- and belowground pathways highlight that borders are complex ecological gatekeepers, simultaneously regulating insect pests, pathogens, and soil-mediated processes. Recognizing this dual influence is critical before moving to management applications, as interventions that target aboveground pests may unintentionally exacerbate belowground pest reservoirs (or vice versa). In the following section, we synthesize these insights to discuss implications for IPM and the design of border plant communities that mitigate pest carryover while maintaining ecosystem services.

## 5. Implications for Pest Management and IPM

The mechanistic pathways outlined above demonstrate that border vegetation is not a peripheral feature of agroecosystems, but an active regulator of pest persistence, colonization, and multitrophic interactions. Yet most integrated pest management (IPM) frameworks remain largely field-centric, emphasizing in-crop interventions such as chemical thresholds, resistant cultivars, crop rotation, and interior cover cropping [6,29,120,121]. Borders are frequently simplified into static landscape elements or categorized dichotomously (e.g., “flower strip” versus “unmanaged margin”), without explicit consideration of their plant composition or functional role [6,28]. This omission can undermine in-field control efforts. If borders maintain alternative hosts, overwintering sites, vectors, or soilborne pathogen reservoirs, pest populations may persist just beyond the crop and re-colonize fields despite effective in-field suppression. Early-season colonization pressure, sequential feeding from weeds to crops, and belowground pathogen carryover illustrate how ignoring border communities creates a structural blind spot in IPM.

Our framework suggests that border management should shift from generalized biodiversity enhancement toward a risk-based strategy grounded in plant identity, functional traits, and seasonal persistence. Pest risk does not emerge simply from plant abundance or species richness, but from the dominance of specific host species (e.g., Figure 2), particularly crop congeners or weeds with overlapping phenology and chemical similarity to crops. A single dominant alternative host can sustain pest populations more effectively than a diverse assemblage of weak or unsuitable hosts (Figure 4), highlighting that plant identity may be more consequential than overall diversity [71,74,122]. Moreover, seasonal continuity is critical: perennial or phenologically synchronized weeds can facilitate pest carryover across cropping cycles, while belowground hosts may maintain nematodes or soilborne pathogens even when aboveground pest presence appears minimal (e.g., [33,80,82,98,107,123]). Effective border management therefore requires identifying high-risk taxa and assessing their dominance structure and temporal persistence, rather than relying on blanket mowing or uniform herbicide application.

Importantly, our synthesis challenges the assumption that increasing border diversity is always universally beneficial. While diverse borders may enhance natural enemy and pollinator communities and disrupt host-finding processes (e.g., [28,30,33,54,57,124]), they may also sustain multiple pest or pathogen reservoirs if crop-associated species are included (Table S1). Diversification should therefore be strategic, not indiscriminate. Composition-based management—potentially guided by diversity thresholds—may offer a more predictive framework. Maintaining sufficient plant richness to prevent host concentration effects, limiting dominance by crop-congeneric or high-risk species, and favoring functionally diverse plants that support natural enemies without serving as key pest hosts represent promising directions. Integrating belowground considerations, such as crop-weed phylogenetic relatedness and plant–soil feedback dynamics, further strengthens this approach.

Taken together, incorporating borders into IPM reframes pest management from a field-centric paradigm to a landscape-connected strategy in which borders function as ecological gatekeepers. Rather than viewing weeds solely as targets for eradication or biodiversity solely as an unquestioned good, a nuanced, composition-driven perspective recognizes that border plant communities regulate pest metacommunity dynamics across spatial and temporal scales. Designing and managing borders with explicit attention to plant identity, seasonal persistence, and belowground interactions offers a pathway toward ecological engineering strategies that balance ecosystem service provision with reduced pest risk.

### Practical Implications for IPM Implementation

Translating these mechanisms into practice requires integrating border vegetation into routine IPM decision-making. We propose several actionable guidelines based on the framework developed here. First, monitoring should extend beyond the crop field to include border vegetation. Identifying dominant plant species, particularly those that are phylogenetically related to crops or known alternative hosts, can provide early warning of pest reservoirs and colonization risk. Second, management should prioritize the selective removal or suppression of high-risk host species rather than indiscriminate vegetation control. Targeting dominant weeds that support pest persistence or overwintering can reduce carryover while maintaining beneficial plant diversity. Third, border design should emphasize functional composition over species richness alone. Incorporating plant species that support natural enemies but are poor hosts for key pests can enhance biological control while minimizing pest reservoirs. Fourth, temporal management is critical. Aligning mowing or disturbance regimes to disrupt pest life cycles—such as before dispersal or reproduction—can reduce spillover into crops without eliminating beneficial habitat. Finally, IPM frameworks should incorporate border conditions into pest risk thresholds and decision-support systems. Accounting for border-mediated colonization pressure and persistence can improve the timing and effectiveness of in-field interventions. Together, these strategies reposition field borders as actively managed components of agroecosystems, enabling more targeted and ecologically informed pest management.

## 6. Future Directions

The framework developed here generates explicit, testable hypotheses that can move border ecology from descriptive surveys toward predictive pest ecology. Several research priorities emerge. First, if borders function as host reservoirs, then pest abundance in early crop stages should increase with the dominance of crop-congeneric or functionally similar weeds in adjacent borders. Experimental manipulation of border composition—particularly dominance structure rather than richness alone—can test whether pest carryover risk is driven by plant identity and seasonal persistence. Second, if borders enhance overwintering success, then pest survival across seasons should correlate with the availability of perennial or phenologically synchronized host species in margins. Mark-recapture approaches, overwintering survival assays, and molecular gut-content analyses could clarify the strength of these spatial and temporal linkages. Third, behavioral spillover hypotheses can be evaluated by testing whether low-diversity, host-dominated borders increase directional pest movement into crops relative to structurally and chemically heterogeneous borders. Movement tracking, volatile manipulation, and visual cue experiments could determine how border sensory environments influence colonization rates. Importantly, these aboveground pathways must be integrated with belowground processes. Future work should examine whether border plant composition predicts soilborne pathogen abundance, nematode persistence, or shifts in microbial communities that alter crop susceptibility. Manipulative plant–soil feedback experiments spanning border and crop soils would help clarify whether belowground reservoirs amplify or buffer pest dynamics. Finally, incorporating border monitoring into routine IPM programs represents a critical applied frontier. Regular assessment of border plant composition, dominance patterns, and high-risk taxa—alongside traditional in-field scouting—could improve prediction of pest outbreaks and inform targeted interventions. Rather than treating borders as static landscape features, monitoring them as dynamic components of pest metacommunities may enhance early-warning capacity and refine management thresholds.

## 7. Conclusions

We acknowledge the importance of field borders for pollinator conservation and integrated pest management. However, borders are not passive margins but active ecological gatekeepers that regulate pest persistence, movement, and interaction networks across agricultural landscapes. By integrating metacommunity theory, sensory ecology, food web dynamics, disturbance filtering, and plant–soil feedbacks, we show that border plant composition—particularly plant identity, dominance structure, and seasonal persistence—shapes both above- and belowground pest pathways.

These findings challenge simplified views of borders as uniformly beneficial or harmful. Instead, pest outcomes emerge from the interaction between plant community structure and ecological processes, highlighting the need for composition-based and risk-informed management. Moving beyond blanket diversification or indiscriminate weed removal, managing borders as dynamic components of agroecosystems offers a pathway toward more predictive and ecologically grounded integrated pest management. Recognizing borders as dynamic nodes within pest metacommunities shifts pest ecology from a field-centric view to a landscape-connected framework, opening new opportunities for ecological engineering and sustainable crop protection.

## Figures and Tables

**Table 1:** Testable predictions for mechanistic pathways linking border vegetation to pest dynamics.

Mechanism	Hypothesis	Testable Predictions
**1 Persistence** (host reservoirs & filters)	Pest persistence depends on temporal host continuity in border vegetation	Pest abundance early in the season increases with the presence and dominance of perennial or off-season host plants in borders; removal or absence of alternative hosts reduces carryover and colonization rates.
**2 Colonization** (behavioral filtering)	Border vegetation structure and composition influence pest movement and host location	Pest colonization rates increase when borders are dominated by crop-like or phenologically synchronized hosts, and decrease with increasing taxonomic and structural diversity that disrupts host-finding cues.
**3 Network restructuring** (multitrophic interactions)	Border vegetation modifies trophic networks, affecting pest suppression outcomes	Increasing plant diversity enhances natural enemy abundance but produces variable pest suppression depending on interaction complexity (e.g., intraguild predation, phenological mismatches, pathogen presence).
**4 Disturbance filtering** (management effects)	Management practices shape border communities and indirectly regulate pest dynamics	High disturbance promotes dominance of fast-growing annual hosts and increases pest spillover; low disturbance promotes perennial vegetation and may increase pest persistence across seasons

## Data Availability

This article has no additional data.

## Supplementary Materials

The following supporting information can be downloaded at https://www.mdpi.com/article/10.3390/biology15090697/s1. Method S1. Quadrat-based plant and pest sampling; Method S2. Literature search and study selection criteria; Table S1. Empirical and conceptual studies included in a structured synthesis of how crop border vegetation influences pest dynamics. References [125,126,127,128,129,130,131,132,133,134,135,136,137,138,139,140,141,142,143,144,145,146,147,148,149] are cited in Supplementary Materials.
