# Supplementary material for: Beyond Beneficial Margins: Four Mechanisms Linking Border Vegetation to Pest Dynamics
Source: Biology (Basel). 2026 Apr 29;15(9):697. doi: 10.3390/biology15090697 (PMC13162615; doi:10.3390/biology15090697)
Supplement: Supplementary file 1 [file biology-15-00697-s001.zip › biology-4237267-supplementary.pdf]

SUPPLEMENTAL MATERIAL

Beyond Beneficial Margins: Four Mechanisms Linking Border Vegetation to Pest Dynamics

Authors: Jorge F. Cardoso[1] & Fabiane M. Mundim[1,2*]

Methods S1. Quadrat-based plant and pest sampling

S1.1 Vegetation sampling design

This study was conducted in northern Utah from 2023 onward across more than a dozen farms; however, only ten farms with complete datasets from 2023 were included in this manuscript. All study systems were Solanaceous cropping systems (e.g., tomato, potato, and eggplant). At each farm, crop fields and adjacent vegetated borders were delineated based on field configuration and vegetation structure. Border habitat was defined following the conceptual distinction in Box 1 as the vegetated strip adjacent to the crop–non-crop interface (hereafter “border”).

We quantified weed diversity in crop and border habitats using quadrat-based photographic sampling. At each sampling location, a 50 × 50 cm quadrat was placed on the ground and photographed from a fixed height of 1 m using a nadir (vertical) orientation to ensure consistent scale and image perspective across all samples.

Within each farm, 15 quadrats were randomly placed in crop interiors and 15 in adjacent border habitats per sampling period (n = 30 quadrats per farm per sampling date). Sampling was conducted three times per growing season (early, mid, and late season). However, because early-season vegetation was often absent or obscured by residual frost or snow cover, only mid-season (July–August) and end-season (October) data are included in this manuscript. These time points capture key stages of vegetation development, including peak growth and senescence, and allow comparison of temporal variation in weed community structure across years.

S1.2 Plant identification and classification

All plant taxa visible within each quadrat image were identified to the lowest possible taxonomic level using standardized botanical identification protocols and reference to Utah State University herbarium collections (https://artsci.usu.edu/herbarium/). A total of 32 taxa has already been collected since the beginning of the study.

When species-level identification was not possible, taxa were classified as morphospecies based on consistent, distinguishable morphological characteristics observable in the images. In the subset of data presented in this manuscript, grasses (Poaceae) that could not be reliably identified to species level were classified at family level due to shared functional roles in agroecosystem structure. Individuals lacking diagnostic morphological traits (e.g., reproductive structures or fully developed vegetative characters) were assigned to unknown morphospecies.

For the dataset analyzed in this manuscript, plant presence was recorded as binary occurrence (0/1) for each taxon within each quadrat. Any rooted individual intersecting the quadrat frame was considered present. Percent cover was not estimated, as the objective was to quantify taxonomic richness and occurrence rather than dominance or biomass. Weed

occurrence was calculated as the proportion of quadrats (15 per habitat per farm per sampling period) in which each taxon was recorded, separately for crop and border habitats and across sampling periods included in this study subset.

The same presence-absence matrix was used for two complementary analytical purposes. First, community-level diversity (Fig. 3) was quantified using Shannon diversity (H′) calculated from quadrat-level taxon frequencies across crop and border habitats. Second, species-level occurrence data were used to compute proportional occurrence and association patterns for a subset of dominant weed taxa and associated pest groups (Fig. 4). This subset-based analysis was used to evaluate plant-pest associations rather than overall community structure.

S1.3 Insect sampling associated with quadrats

In this manuscript, insect–plant associations were assessed by sampling insects within each quadrat and in the immediately adjacent vegetation using complementary collection methods. A handheld vacuum sampler fitted with a fine mesh collection bag was used to capture small and cryptic taxa. Sweep netting was then applied to collect larger and more mobile insects to ensure broader sampling coverage across functional groups.

All specimens were transferred to labeled collection bags indicating farm, habitat type (crop or border), quadrat ID, sampling period (mid- or end-season), and date. Samples were stored in cooled containers during fieldwork and subsequently transported to the laboratory for identification using reference collections.

For this manuscript, insect data were used in two complementary ways. First, all collected arthropods were used to characterize general pest presence associated with crop and border vegetation. Second, Fig. 4 focuses on a subset of visually detectable pest taxa for which consistent associations with dominant weed species could be documented across sampling periods. This subset-based approach was used to visualize consistent plant–pest associations rather than to estimate absolute pest abundance.

S1.4 Plant pathogen assessment

Plants within each quadrat were visually inspected for symptoms consistent with fungal or viral infection, including chlorosis, necrotic lesions, mosaic patterns, deformation, and other abnormal growth abnormalities. Symptomatic plants were collected, labeled, and transported to the laboratory for confirmation and taxonomic identification of associated pathogens when possible. For this manuscript, pathogen observations were used to document the occurrence of disease symptoms associated with dominant weed species within crop and border habitats. These records were integrated with plant occurrence data to support the association-based synthesis presented in Fig. 4. This analysis is intended to describe co-occurrence patterns between weed taxa and pathogen presence rather than quantify pathogen incidence or infection rates.

S1.5 Soil sampling for nematodes

Soil samples were collected within each quadrat to assess the presence of plant-parasitic nematodes. After removing surface debris, soil was collected from a depth of approximately 10–20 cm within the root zone of representative plants, particularly those associated with plant-parasitic nematodes such as *Meloidogyne* spp.. Samples were placed in labeled plastic bags, kept cool during transport, and subsequently processed in the laboratory for nematode extraction and taxonomic identification. For this manuscript, nematode records were used as part of an association-based synthesis linking weed taxa to belowground pest presence across crop and border habitats. These data contribute to the multitrophic interaction framework presented in Fig. 4 and are not intended to represent exhaustive estimates of nematode density or field-wide infestation levels.

Methods S2. Literature search and study selection criteria

To evaluate how border weed communities are represented in the agricultural pest ecology literature, we conducted a structured keyword search in Google Scholar using the following Boolean string:
(“field margin” OR “crop border” OR “field edge” OR “non-crop vegetation” OR “edge habitat” OR “vegetated margin”) AND (weed* OR “weed community” OR “wild plant*” OR “spontaneous vegetation” OR “border vegetation”) AND (“alternative host*” OR overwinter* OR “pest reservoir*” OR spillover OR colonization OR “pest persistence” OR “population dynamics”).

The search returned 4,680 records. No temporal restriction was applied.
All records were screened through full-text review. Studies were included in the synthesis if they met all four criteria:

1. The study was conducted in an agricultural context (crop systems, agroecosystems, or farmed landscapes).
2. The study explicitly examined border, margin, or edge vegetation containing weed or spontaneous plant communities.
3. The study identified at least one pest, pathogen, or beneficial organism associated with border vegetation.
4. The study results could be interpreted within at least one of the four mechanistic pathways defined in the main text (Section 3).

Both empirical and conceptual (review) studies were eligible for inclusion. Studies not meeting all four criteria were excluded from Table S1 and Fig. 2.

S2.2 Mechanism classification procedure

Each included study was assigned to one or more mechanistic categories based on its primary ecological inference. Classification was interpretive and based on explicit evidence or clear functional inference in the text:

- Fig 5a and Table 1: Host reservoirs and selective filters: studies reporting overwintering, alternative host use, or seasonal persistence of pests in border vegetation

- Fig 5b and Table 1: Colonization and behavioral modification: studies addressing pest movement, host-finding, spillover, or edge-mediated dispersal
- Fig 5c and Table 1: Trophic and disease network restructuring: studies reporting interactions involving natural enemies, pathogens, alternative prey, or multitrophic effects
- Fig 5d and Table 1: Management as a selective force: studies linking disturbance, mowing, herbicide use, or habitat management to changes in border vegetation and pest outcomes

Multiple assignments were allowed when studies reported evidence spanning more than one pathway (see Fig. 2 and Table S1)

This approach prioritizes mechanistic interpretability over exhaustiveness, consistent with the conceptual aim of the synthesis.

Supplemental Table

Table S1. Empirical and conceptual studies included in a structured synthesis of how crop border vegetation influences pest dynamics. Synthesis of empirical and review studies evaluating how vegetated field borders influence pest populations, natural enemies, and associated weed communities in agroecosystems. Studies were identified through a targeted and structured literature survey of peer-reviewed empirical and conceptual work addressing field borders, field margins, or vegetated strips in agroecosystems. Studies were included if they explicitly examined effects of border vegetation on pest populations, natural enemies, or associated trophic interactions and could be interpreted within at least one of the four mechanistic pathways proposed in this framework (see Supplemental Material for literature review search criteria). The classification is interpretive and intended to organize existing evidence rather than provide an exhaustive systematic review. Studies were categorized according to four mechanistic pathways: (1 Persistence) host reservoirs and selective filters; (2 Colonization) modification of pest behavior and colonization; (3 Network Restructuring) restructuring of trophic and disease dynamics; and (4 Disturbance) management as a selective force. Multiple mechanisms may apply to a single study. Target organism refers to the focal taxonomic or functional group, and inferred role of border summarizes the functional interpretation of border vegetation effects.

Author	Year	Target organism	Mechanistic pathways linking border weeds to plant pest ecology	Inferred role of Border
Štefanić E. [1]	2020	arable weeds	1	Reservoir (weeds); colonization modulator
Petit et al. [2]	2023	Carabid beetles (weed herbivores)	3	Reservoir (natural enemies); multitrophic restructurer
Schnee et al. [3]	2023	Arable weeds (e.g., *Chenopodium album*, *Rumex crispus*)	1; 4	Reservoir (weeds); management filter
Mkenda et al. [4]	2019	Insect pests, natural enemies, pollinators (conceptual/review)	1; 2; 3; 4	Reservoir (pests & enemies); multitrophic restructurer; management filter
Mkenda et al. [5]	2019	Aphids predators and parasitoids	1; 3	Reservoir (natural enemies); colonization modulator; multitrophic restructurer

Tscharntke et al. [21]	2005	Crop pests; natural enemies	2; 3; 4	Colonization modulator; multitrophic restructurer
Marshall & Moonen [30]	2002	Arable plants & arthropods	3; 4	Multitrophic restructurer; management filter
Marshall [31]	2004	Aphid, weevil and mollusk Pests (conceptual/review)	1; 2; 4	Reservoir (pests & pathogens); colonization modulator; management filter
Bianchi et al. [35]	2006	Natural enemies & pests	3	Multitrophic restructurer (landscape-mediated)
Barbercheck & Wallace [37]	2021	Insect Pests and natural enemies (conceptual/review)	1; 2; 3; 4	Reservoir (pests & enemies); colonization modulator; multitrophic restructurer; management filter
Bischoff et al. [48]	2016	Beetle, aphid, fly pests	1; 2; 3	Reservoir (pests); multitrophic restructurer
Pollier et al. [49]	2018	Insect pests, natural enemies	1; 2; 3	Reservoir (pests & enemies); multitrophic restructurer
Mundim et al. [52]	2026	Parasites & pathogens (conceptual/review)	1; 3	Reservoir; disease network restructurer
Bàrberi et al. [64]	2010	Insect pests (conceptual/review)	1; 2; 3; 4	Reservoir (pests & enemies); multitrophic restructurer; management filter
Grafius & Collins [75]	1986	Carrot weevil	1; 2; 4	Reservoir (pests); colonization modulator; management filter

Babu et al. [76]	2019	Brown stink bug	1; 4	Reservoir (pests); management filter
Larentzaki et al. [77]	2007	Onion thrips	1; 2; 4	Reservoir (pests); management filter
Silva et al. [78]	2018	Onion thrips, Western flower thrips, Cotton thrips	1; 2; 4	Reservoir (pests); colonization modulator
Sybilska et al. [79]	2025	Tomato russet mite (*Aculops lycopersici*)	1; 2; 4	Reservoir (pests); colonization modulator
Andersen et al. [80]	2005	Crucifer flea beetle	1; 2	Reservoir (pests); colonization modulator
Mueller et al. [81]	2012	Alfalfa mosaic virus (AMV) and Cauliflower mosaic virus (CaMV) vectores by *Aphis glycines*	1	Reservoir (pathogens); colonization modulator
Losey et al. [82]	2001	European corn borer	1; 2	Colonization modulator
Esquivel et al. [87]	2020	Cotton fleahopper; Verde plant bug	1; 2; 4	Reservoir (pests); colonization modulator
Nault et al. [88]	1997	Colorado potato beetle (*Leptinotarsa decemlineata*)	1; 2	Colonization modulator
Ndakidemi et al. [89]	2022	Aphids pests and natural enemies	2; 3; 4	Reservoir (natural enemies); colonization modulator; multitrophic restructurer; management filter
Van Alebeek et al. [93]	2004	Aphid pests, ground beetles (predator), spiders (predator)	1; 3; 4	Reservoir (natural enemies); multitrophic restructurer

Dennis & Fry [94]	1992	Aphid predators	1; 3; 4	Reservoir (natural enemies); multitrophic restructurer
Völkl & Stechmann [95]	1998	Aphid pests	1; 2; 3	Reservoir (pests & enemies); colonization modulator; multitrophic restructurer
Haaland et al. [96]	2011	Insect Pests, natural enemies, pollinators (conceptual/review)	2; 3; 4	Reservoir (beneficials); multitrophic restructurer; management filter
Jowett et al. [97]	2024	Carabid beetles (predator)	2; 3; 4	Multitrophic restructurer; management filter
Lagerlöf & Wallin [102]	1993	General crop arthropods (Predators, herbivores and parasitoids)	1; 3; 4	Reservoir (natural enemies); multitrophic restructurer; management filter
Thomas & Marshall [103]	1999	Carabid beetles (predator), insect pests	1; 3; 4	Reservoir (beneficials); multitrophic restructurer; management filter
Weaver et al. [105]	2004	Wheat stem sawfly and parasitoid natural enemies	1; 2; 4	Colonization modulator; multitrophic restructurer; management filter
Pei et al. [107]	2024	Meloidogyne enterolobii	1	Reservoir (pests); colonization modulator
Letourneau et al. [122]	2011	Crop pests; natural enemies (review)	3	Multitrophic restructurer (meta-analysis)
da Silva Brito et al. [124]	2021	Insect pests, natural enemies, decomposers	2; 3	Colonization modulator; multitrophic restructurer

Lewis [125]	1969	Pollen beetles (Brassicaceae pests) Magpie moth (*Abraxas grossulariata)* and aphids	1; 2	Reservoir (pests); colonization modulator
Fye [126]	1980	Lygus hesperus, *L. elisus*	1; 4	Reservoir (pests); colonization modulator; management filter
Jones & Sullivan [127]	1982	*Nezara viridula, Acrosternum hilare, Euschistus servus*	1; 2	Reservoir (pests); colonization modulator
Lethmayer et al. [128]	1997	Coleopteran crop pests of Families Nitidulidae, Chrysomelidae and Curculionidae	1; 2; 4	Reservoir (pests); management filter
Thomas et al. [129]	1998	Carabid beetles	1; 3	Reservoir (natural enemies); multitrophic restructurer
Landis et al. [130]	2000	Natural enemies (review)	1; 3	Reservoir (beneficials); multitrophic restructurer
Denys & Tscharntke [131]	2002	Insect herbivores on experimental plant patches (e.g., clover herbivores) and natural enemies (mainly spiders); focus on colonization + predator:prey structure	2; 3; 4	Reservoir (natural enemies); colonization modulator; management filter
Meek et al. [132]	2002	None (biodiversity study)	3	Multitrophic restructurer
Thomas et al. [133]	2002	Carabid beetles	2; 3	Reservoir (natural enemies); colonization modulator; multitrophic restructurer
Thomas [134]	2005	Insect Pests, natural enemies, pollinators (conceptual/review)	1; 3; 4	Reservoir (pests & enemies); multitrophic restructurer; management filter

Frank & Künzle [135]	2006	*Lygus pratensis, Lygus rugulipennis, Eurydema oleracea, Dicyphus globulifer, Plagiognathus arbustorum, Adelphocoris lineolatus, Orthotylus flavosparsus, Stictopleurus punctatonervosus*	3; 4	Reservoir (beneficials); multitrophic restructurer; management filter
Rand et al. [136]	2006	Arthropods in field margins	2; 3	Colonization modulator; multitrophic restructurer
Tscharntke et al. [137]	2007	Multiple trophic levels	1; 3	Reservoir; multitrophic restructurer
Haddad et al. [138]	2009	Trophic networks	3	Network restructurer
Fox et al. [139]	2013	Field crickets, ground beetle, fire ant (weed herbivores)	3; 4	Multitrophic restructurer; management filter
Molina et al. [140]	2013	Insect pests (conceptual/review)	1; 2; 3; 4	Reservoir (pests & enemies); colonization modulator; multitrophic restructurer
Blaauw & Isaacs [141]	2014	Natural enemies; pests	2; 3	Multitrophic restructurer; colonization modulator
Pywell et al. [142]	2015	Pollinators; crop yield	3; 4	Multitrophic restructurer; management-driven filter
Holland et al. [143]	2016	Carabids; pest suppression (review)	3	Reservoir (beneficials); multitrophic restructurer
Gurr et al. [144]	2017	Pests & biological control agents	3; 4	Multitrophic restructurer; management filter

**Terfa** [145]	2018	Viruses, insect and nematode pests	1; 4	Reservoir (pests & pathogens); management filter
**Haan et al.** [146]	2020	Arthropods in margins	3; 4	Multitrophic restructurer; management filter
**Canovas et al.** [147]	2023	*Frankliniella spp*	1; 2	Reservoir (pests); colonization modulator
**Tóth et al.** [148]	2023	Pests of genus Nysius, Lygus, and other true bugs; Natural enemies of genus Nabis	1; 2; 3	Reservoir (pests & pathogens); colonization modulator
**Dorner et al.** [149]	2024	Ground Beetles (weed herbivores)	1; 3	Reservoir (natural enemies); multitrophic restructurer
